# Supplementary figures and images for: Risk Assessment of Transgender People: Development of Rodent Models Mimicking Gender-Affirming Hormone Therapies and Identification of Sex-Dimorphic Liver Genes as Novel Biomarkers of Sex Transition
Source: Cells. 2023 Feb 1;12(3):474. doi: 10.3390/cells12030474 (PMC9913858; doi:10.3390/cells12030474)

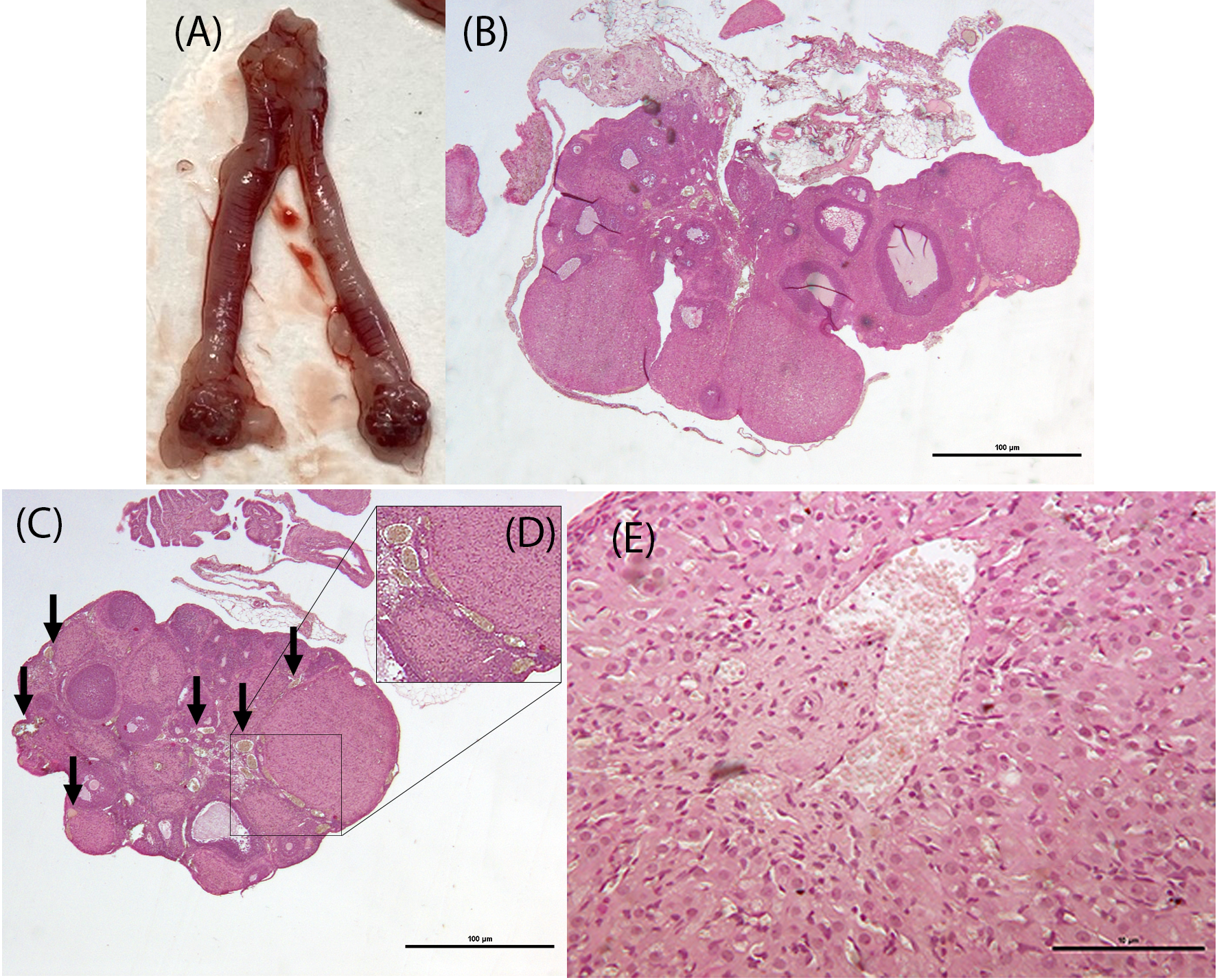

Supplement: Supplementary file 1 [file cells-12-00474-s001.zip › cells-2067739-supplementary.tif]
